# Draft genome sequence of an entomopathogenic fungus *Amphichorda felina*

**DOI:** 10.1128/mra.01125-25

**Published:** 2026-02-24

**Authors:** Ngoc-Hung Nguyen, Phuong-Thao Nguyen, Toshimitsu Fukiharu, Haruka Kameda, Kiminori Shimizu

**Affiliations:** 1 Department of Computational Biology and Medical Sciences, Graduate School of Frontier Sciences, University of Tokyo 105219 , Kashiwa, Chiba, Japan; 2 Department of Biological Science and Technology, Tokyo University of Science 378550 , Tokyo, Japan; 3 Natural History Museum and Institute, Chiba, Japan; 4 Graduate School of Human and Environmental Studies, Kyoto University 12918, Kyoto, Japan; 5 Medical Mycology Research Center, Chiba University 12737, Chiba, Japan; University of Strathclyde, Glasgow, United Kingdom

**Keywords:** entomopathogen

## Abstract

An entomopathogenic fungus, *Amphichorda felina* (formerly classified as *Beauveria felina*), was sequenced through Illumina MiSeq short reads and Oxford Nanopore long reads. The hybrid assembly resulted in a genome of approximately 29.6 Mb with 9 scaffolds and an N50 of 3.28 Mb.

## ANNOUNCEMENT

*Amphichorda felina* (formerly *Beauveria felina*) is an entomopathogenic fungus with biocontrol potential. Recent multi-locus phylogenetic analyses revealed that this species is distinct from other *Beauveria* species, leading to its reclassification into *Amphichorda* (Bionectriaceae, Sordariomycetes, Hypocreales) (1). To support future biocontrol applications, we sequenced and assembled the genome of *A. felina* strain CBM-FB-45539.

The wild strain CBM-FB-45539 was isolated from deer feces collected on 4 December 2021 on Yakushima Island, Japan. Dung was incubated in a moist chamber (1,000 mL bottle with sterilized, moistened moss) at 22°C under a 12 h light/dark cycle (2). After two weeks, basidiomata developed; a fragment was aseptically transferred to MYC medium to establish CBM-FB-45539. The strain was deposited in the Natural History Museum and Institute, Chiba, Japan (CBM-FB-45539) and maintained on MYC agar at 4°C until DNA extraction. Mycelia from 7-day cultures were harvested, and DNA was extracted separately for MGI and Nanopore sequencing using the DNeasy Plant Mini Kit (Qiagen) following the manufacturer’s protocol. Taxonomic status was confirmed by ITS rDNA sequencing (primers ITS1/ITS4 [3]; GenBank PV413099.1). BLASTn against NCBI nt (accessed 8 December 2022) showed 98.93% sequence identity to *A. felina* strain CBS 250.34 (GenBank AY261369.1), and monophyly was confirmed by phylogenetic analysis (4).

Illumina libraries were prepared using the MGIEasy PCR-Free DNA Library Prep Set and sequenced on DNBSEQ-G400RS (paired-end 150 bp). Nanopore DNA was prepared without shearing or size selection using the Native Barcoding Kit 24 V14 (SQK-NBD114.24) and sequenced on PromethION (FLO-PRO114M flow cells). Base calling was performed using Dorado v0.7.3 with the SUP model. We obtained 36,771,966 MiSeq read pairs (~9.79 Gb) and 361,240 long reads (2.86 Gb). Long reads were adapter-trimmed (Porechop v0.2.4), contamination-filtered (NanoLyse v1.1.0 [5]), and size-filtered >1,000 bp (SeqKit v2.2.0 [6]). Illumina reads were quality-filtered and adapter-trimmed (cutadapt v4.2 [7]), retaining reads with Phred quality >20 and minimum length of 20 bp.

The mitochondrial genome was assembled and circularized from Illumina reads using GetOrganelle v1.7.7.1 (8), which automatically detects and trims overlapping terminal regions. Reads from both platforms were mapped to the mitochondrial genome using Minimap2 v2.22 (9), and mitochondrial reads were removed (samtools v1.16.1 [10]). The nuclear genome was assembled from remaining reads using MaSuRCA v4.1.2 (11), polished by mapping MiSeq reads (BWA-MEM v0.7.17 [12]) followed by four rounds of Pilon v1.24 (13). The mitochondrial genome was annotated using MFannot (14) and MITOS2 v2.1.3+galaxy0 (15) on Galaxy (16) (Genetic Code 4); both resulting annotations were inspected in IGV (17) and reconciled by manual curation to produce a single final mitochondrial genome annotation. Genome completeness was assessed using BUSCO v5.4.3 with 3,817 Sordariomycetes orthologs (18). Repeats were predicted (RepeatModeler v2.0.1 [19]) and masked (RepeatMasker v4.1.1 [20]). The Funannotate v1.8.1 pipeline (21) predicted 9,427 protein-coding genes across the nine assembled contigs (Table 1). The complete mitochondrial genome is a circular molecule of 27,301 bp (single closed contig) containing 43 genes: 2 rRNA, 17 protein-coding, and 24 tRNA genes. Default parameters were used unless otherwise specified.

**TABLE 1:** Genome features of the *Amphichorda felina* assembly and annotation

Characteristic	Value
Chromosome contigs	
Total assembled length (bp)	29,588,262
No. of contigs	9
N50 (bp)	3,278,244
L50	4
Max length (bp)	4,728,797
No. of complete BUSCOs (%)	3,747 (98.1%)
No. of incomplete BUSCOs (%)	6 (0.2%)
No. of missing BUSCOs (%)	64 (1.4%)
Repeats	1.74%
GC	54.68%
No. of protein-coding genes	9,270
No. of tRNAs	157
No. of Phobius secretome genes	1,109
No. of Phobius transmembrane proteins	2,018
No. of antiSMASH biosynthetic gene clusters	49
Mitochondrial genome	
Length (bp)	27,301
No. of protein-coding genes	17
No. of rRNAs	2
No. of tRNAs	24

## Data Availability

The Amphicorda felina genome project, including raw Illumina and Nanopore data, has been deposited at DDBJ/EMBL/GenBank under BioProject accession number PRJNA1174440. The raw MiSeq and Nanopore sequencing reads have been deposited at the Sequence Read Archive under accession numbers SRR31037621 and SRR31037620, respectively. The assembled nuclear genome and assembled mitochondrial sequences are available under GenBank accession numbers JBIMJW000000000 (the version described in this paper is version JBIMJW010000000.1) and PQ516710.1, respectively.
